# Neural markers of social dominance: A female-focused perspective

**DOI:** 10.1016/j.isci.2025.113109

**Published:** 2025-07-15

**Authors:** Wei-Hsiang Lin, Janir Ramos da Cruz, Carmen Sandi, Michael H. Herzog

**Affiliations:** 1 Laboratory of Psychophysics, Brain Mind Institute, School of Life Sciences, Swiss Federal Institute of Technology Lausanne (EPFL), 1015 Lausanne, Switzerland; 2 Laboratory of Behavioral Genetics, Brain Mind Institute, School of Life Sciences, Swiss Federal Institute of Technology Lausanne (EPFL), 1015 Lausanne, Switzerland; 3 Wyss Center for Bio and Neuroengineering, Geneva, Switzerland

**Keywords:** neuroscience, behavioral neuroscience, cognitive neuroscience

## Abstract

Social interactions are fundamental to human life, with social dominance being a key factor in these interactions. Previous studies have shown that dominant males are faster in decision-making tasks compared to non-dominant ones, even in the absence of a social context such as competition. Additionally, dominant males exhibit a significantly higher N2/P2 EEG component, which is an inherent trait rather than a state marker of dominance. While it has been suggested that social hierarchies are more pronounced among males, recent findings challenge this notion. Here, we show that the N2/P2 component is also higher in dominant than in less dominant females, with similar amplitude and latency as their male counterparts. Our results suggest that women exhibit dominance-related neurobiological traits similar to men. Our findings underscore the importance of further investigating the socio-cultural and environmental factors that contribute to gender disparities in social hierarchies.

## Highlights

• Social dominance is a stable personality trait
• Specific EEG components serve as reliable neural markers of dominance
• Neural dominance markers in females mirror those found in males

## Graphical abstract

**Figure:**
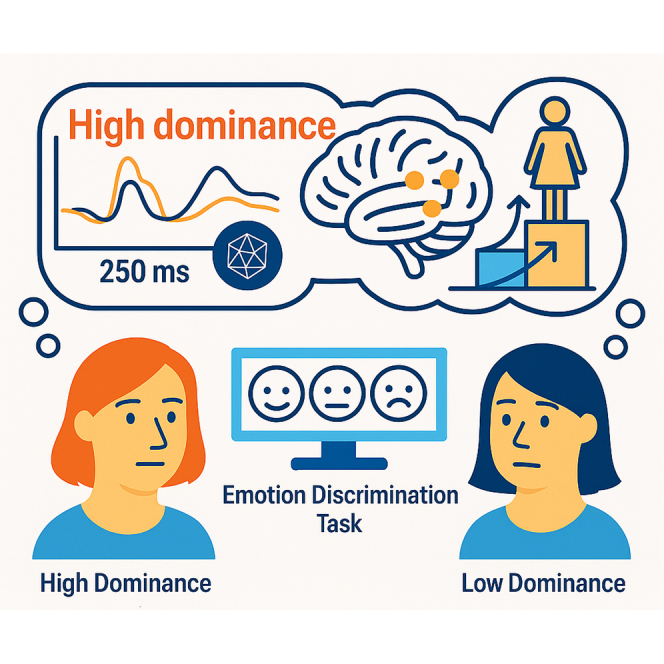

## Introduction

Social dominance is a fundamental component of social groups, often manifesting in the deep hierarchical structures that are observed.^1^^,^^2^

Classically, social dominance has been investigated in paradigms where, for example, two or more participants compete for different ranks, and performance becomes the criterion for determining the dominance level. A key insight from these studies is that social dominance is a relative scale, fundamentally relying on comparisons with others, for instance, the comparison within a social group.^3^

However, several studies have suggested that social dominance is a distinct personality trait.^4^^,^^5^ Dominant individuals often exhibit characteristic behaviors, including extended speaking durations^6^ and a higher perception of competence.^7^ These findings imply that social interactions might not be a prerequisite for assessing social dominance. This hypothesis received support from a previous study,^8^ where dominant male participants were faster than less dominant males in a complex decision-making task that involved no social context. In addition, the dominant males had a much higher N2/P2 EEG component. This finding indicates that dominant males allocate more resources when making decisions.^9^

Social dominance is predominantly studied in male participants.^8^^,^^10^ While some studies have suggested an interplay between levels of social dominance and gender in stress behavior, both in animals^11^ and humans,^12^ and in political attitudes,^13^ other research, such as that by Scheggia et al. (2022),^14^ reports no gender differences in social-decision-making scenarios. Here, we adopted the procedure from da Cruz et al. (2018)^8^ and show that females exhibit the same N2/P2 EEG component as males do.

## Results

### Behavioral results

**Figure 1:**
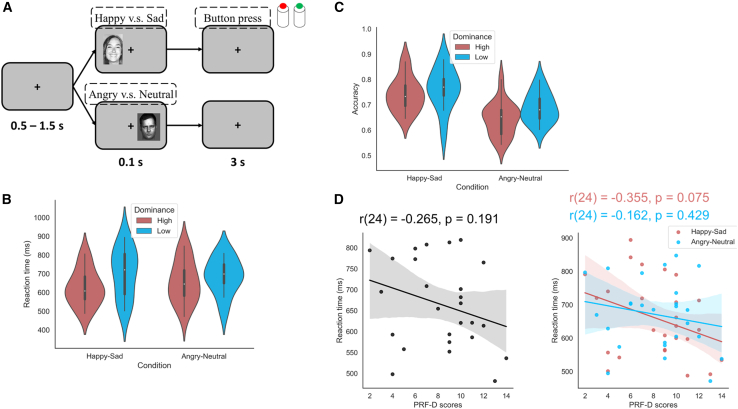
Behavioral results in the emotion discrimination task (A) Participants discriminated between happy and sad or angry and neutral faces. They were required to push a corresponding button as accurately and as quickly as possible. (B) Violin plots showing the reaction times of the high and low dominance groups in the two experimental conditions. Main effect of dominance group on reaction time (F(1,22.41) = 2.442, *p* = 0.132, partial η2 = 0.098). (C) Violin plot showing the accuracy of both the high and low dominance groups in the two experimental conditions. Main effect of dominance group on accuracy (F(1,21.4) = 1.575, *p* = 0.223, partial η2 = 0.068). (D) Scatterplot displaying the correlation between PRF-D scores and reaction times. Violin plots in (B) and (C) illustrate the data distribution, with horizontal lines marking the median and quartiles.

Dominance was assessed using the dominance scale of the personality research form (PRF-D^15^). Participants with scores greater than or equal to the median were assigned to the high dominance group, and those with scores less than the median were assigned to the low dominance group. In the experiment, participants discriminated between a happy and a sad face or between an angry and a neutral face (Figure 1A), as it was in da Cruz et al. (2018).^8^ Here, we found only a trend between dominance levels and reaction times (Figure 1D; r(24) = −0.355, *p* = 0.075) and no effect on the group level (Figure 1B; F(1,22.41) = 2.442, *p* = 0.132, partial η2 = 0.098). There was no main effect of dominance on accuracy (Figure 1C; F(1,21.4) = 1.575, *p* = 0.223, partial η2 = 0.068). There was no significant effect on reaction times (F(1,20.52) = 0.122, *p* = 0.730, partial η2 = 0.006).

Previous studies showed that testosterone is positively correlated with dominance, but only in individuals with low levels of cortisol.^16^ Here, we found no significant differences between the two groups (Figure S1).

### EEG results

**Figure 2:**
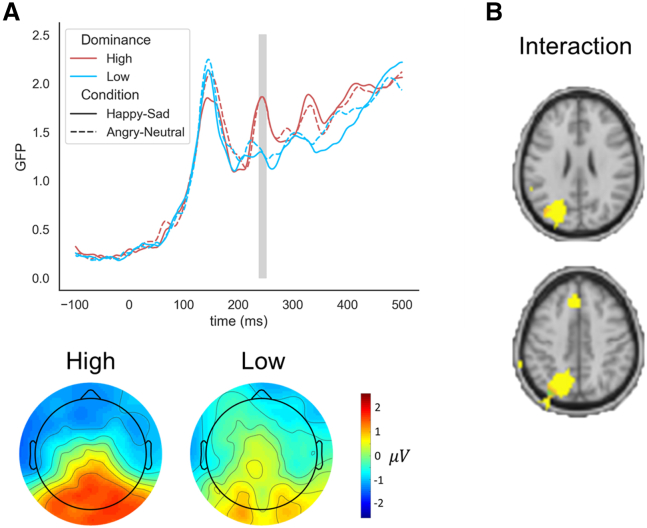
High dominant females exhibit an elevated N2/P2 EEG component (A) Top: GFP traces for the two groups in the happy vs. sad and angry vs. neutral experimental conditions. The gray region marks the time points that show a main effect of group. Bottom: averaged topographies of the two groups. (B) The interaction effect of groups and conditions in the source space. Voxel-based repeated measures ANOVA performed at each voxel yielded a significant interaction effect for the dominance groups comparison at specific voxels (yellow voxels). The two images (top and bottom) represent the same map at different depths.

We recorded EEG signals while participants performed the emotion discrimination task. Global field power (GFP) was computed as the standard deviation of all electrodes. Dominant females had significantly higher N2/P2 amplitudes than the less dominant ones at 253 ms after stimulus presentation (Figure 2A), mirroring the results from da Cruz et al. (2018)^8^ with male participants.

We performed a source localization analysis and performed repeated measures ANOVA to each of the voxel in the source space with the effect of dominance (high vs. low) and the experimental conditions (happy-sad vs. angry-neutral). We found an interaction effect in the activity of the left intraparietal sulcus (IPS): similar activity was observed between the two dominance groups in the happy-sad condition, while higher activity in dominant females in the angry-neutral condition (Figure 2B). In the happy-sad condition, we found, in addition to the IPS, differences between the groups in the striatum and the insula (Figure S2A). Conversely, in the angry-neutral condition, differences were associated with the temporal regions (Figure S2B).

**Figure 3:**
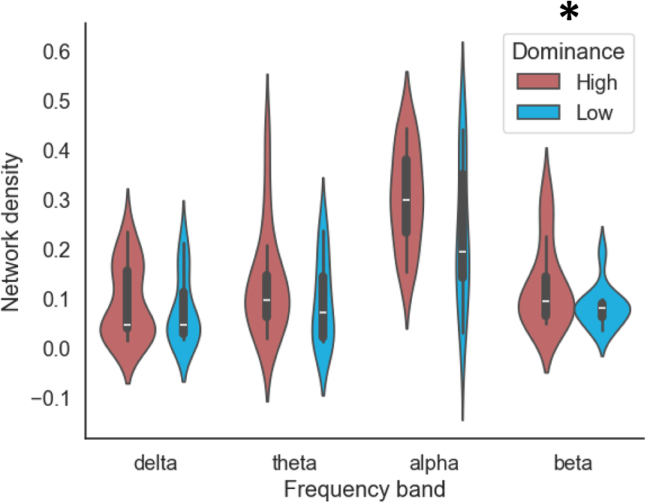
Social dominance and resting state EEG The network density in the two groups for four frequency bands. The asterisk indicates a significant main effect of the dominance group (repeated measures ANOVA, *p* < 0.05). The violin plot illustrates the data distribution, with horizontal lines marking the median and quartiles. Main effect of dominance group on network density (F(1,24) = 4.413, *p* = 0.046, partial η2 = 0.155).

The N2/P2 differences were observed even in the absence of a social context but still a task was performed. To test whether we can find even task-independent markers for social dominance, we measured resting-state EEG after the above experiment. EEG was recorded during 5 min where participants just relaxed with their eyes closed. Whole-brain network density, which was computed from the connectivity analysis, revealed a main difference between the two dominance groups (Figure 3; F(1,24) = 4.413, *p* = 0.046, partial η2 = 0.155).

### Control task results

**Figure 4:**
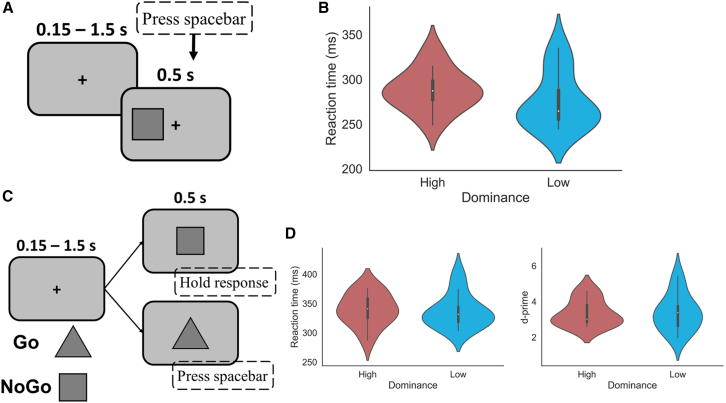
Simple reaction time and Go/No-Go task (A) Participants pressed the spacebar as soon as they noticed the onset of a square on the screen. (B) Violin plots showing the reaction times of both the high and low dominant groups. No significant difference in reaction times was observed between the two dominance groups (t(24) = 1.309, *p* = 0.203, Cohen’s d = 0.52). (C) Go or No-Go stimuli appeared on the screen. Participants were required to press the spacebar when a Go stimulus was presented and withhold their response when a No-Go stimulus was presented. (D) Left: violin plot showing the reaction times of hit trials for the high and low dominant groups. Right: violin plot showing the d-prime scores of both high and low dominant groups. No significant differences in reaction times (t(24) = 0.866, *p* = 0.395, Cohen’s d = 0.344) or d-prime scores (t(24) = −0.419, *p* = 0.679, Cohen’s d = 0.167) were observed between the two dominance groups. Violin plots in (B) and (D) illustrate the data distribution, with horizontal lines marking the median and quartiles.

There was a trend for faster reaction times in the dominant females. Next, we tested whether these faster reaction times just come from faster motor skills by using a classic speeded reaction time task (Figure 4A). No significant differences emerged in the reaction times between the two dominance groups, suggesting comparable motor skills (Figure 4B; t(24) = 1.309, *p* = 0.203, Cohen’s d = 0.52). We further examined potential differences in impulsivity between the groups by conducting a Go/No-Go task (Figure 4C), in which participants were required to respond quickly to a predefined Go stimulus while inhibiting the response to a NoGo stimulus. There were neither significant differences in the reaction times during hit trials (t(24) = 0.866, *p* = 0.395, Cohen’s d = 0.344) nor in the d-prime scores (t(24) = −0.419, *p* = 0.679, Cohen’s d = 0.167) between the groups (Figure 4D).

## Discussion

The N2/P2 is strongly elevated in dominant males compared to less dominant ones.^8^ We found a very similar result in females, with the peak amplitude occurring at a similar magnitude and at the same time, around 230 ms. Hence, this characteristic marker of social dominance, observed while individuals perform decision-making, appears to be similar in both males and females.

The N2/P2 component is related to different aspects of the decision-making process, such as attention allocation,^17^ target categorization,^9^ and intrinsic efforts, which may all be stronger in dominant males and females, compared to less dominant individuals. We performed source localization to identify the brain regions contributing to the differences in the N2/P2 EEG component. We found significant differences in the IPS, the insula, the striatum, and the temporal sulcus, whereas in males, we previously found differences only in the insula. Our results are in line with a previous study that also showed increased activity in the IPS for dominant females in an emotion discrimination task using similar stimuli as those used in this study.^18^ Thus, dominant females may be able to activate increased resources, which then lead to the enhanced N2/P2 component. We would like to note that both the striatum and the intraparietal sulcus (IPS) are involved in encoding social status.^19^^,^^20^^,^^21^^,^^22^^,^^23^ However, since we did not find corresponding results in the male population, these considerations remain speculative.

Social dominance has been mainly studied with the involvement of tasks^8^^,^^10^^,^^23^ Here, we analyzed resting-state EEG data to determine whether there are significant differences between dominant females even without any task. We found a significant difference in connectivity density, with the high dominance group having denser connectivity compared to the low dominance group. Hence, it seems that dominance is reflected in many brain features, potentially reflecting the importance of dominance in human life. The observation that high-dominant individuals exhibit higher network density in resting-state EEG suggests that their neural architecture may be more integrative, allowing for a greater flow of information across distributed brain regions.^24^^,^^25^ On one hand, this higher density could facilitate more efficient communication between regions that underlie social behaviors—potentially enhancing top-down control, rapid decision-making, and adaptive responses in complex social settings.^26^ At the same time, however, extensive connectivity may reduce the specialization of certain subnetworks, resulting in less modularity or segregation.^26^^,^^27^ In the context of social dominance, this trade-off might be advantageous if broad, integrative neural coordination supports the cognitive and affective demands of asserting dominance.^23^ Nevertheless, determining whether these denser networks primarily cf. benefits for social behavior or potentially limit the flexibility of specialized processing is an important direction for future research.

Dominant males respond faster in complex decision-making paradigms compared to subordinate ones.^8^^,^^28^ We did not find a significant reaction time benefit for high versus low dominance in females but only a trend. This discrepancy may be sex-specific or may come from differences in the sampling methods in the two studies. da Cruz et al. (2018)^8^ used an extreme sampling approach, selecting participants from the two ends of the dominance spectrum (very high and very low). Our study utilized a random sampling method, resulting in a normally distributed range of dominance levels among participants. The observed modulation of N2/P2 signals suggests that dominance perceptions influence the neural processing of emotional stimuli.^29^^,^^30^ However, the absence of corresponding behavioral changes highlights a complex relationship between neural activation and overt behavior. One plausible explanation for the lack of behavioral alterations lies in socio-cultural influences that govern gender-specific expressions of dominance.^31^ These cultural constraints could act as a moderating factor, allowing neural signatures of dominance to persist while limiting their behavioral manifestations. These findings align with social dominance theory,^3^ which posits that dominance hierarchies are maintained through both subtle neural mechanisms and overt social behaviors.^3^ The presence of neural signatures without behavioral changes suggests that dominance may be internally regulated and context-dependent, with socio-cultural factors playing a pivotal role in shaping its external expression.

Previous studies suggested that social dominance may be positively linked to impulsivity.^32^ Here, we found no differences in reaction times in the Go/No-Go task and the simple reaction time task. This implies that the differences we observed might be related to complex decision-making process rather than action execution or cognitive control.

In conclusion, we identified neuromarkers of social dominance in females during decision-making, similar to the observations made by da Cruz et al. (2018)^8^ in males. Since social dominance is often associated with leadership, our findings suggest that females are as “ready” for leadership as males.

### Limitations of the study

One limitation of the current study is that we used only students as participants. It would be valuable to include individuals with more real-life experience, such as leaders. On the other hand, it is surprising to find such clear-cut EEG markers between dominant and less dominant participants even in a rather homogeneous student sample and simply by applying median splits. We are expecting even larger effects in the general population, in which social hierarchies are a much stronger reality. Furthermore, the PRF-D questionnaire we used is somewhat limited, as it comprises only 16 items and thus may capture only certain aspects of the dominance trait. To gain a more comprehensive perspective on social dominance, future studies could combine the PRF-D with additional questionnaires that assess a broader range of dominance-related behaviors and traits. In addition, the choice of reference in EEG studies can influence the interpretation of neural data. While the average reference is widely used, it assumes that the integral of surface potentials over the scalp approximates zero, an assumption that may not hold true due to the complex shape of the human head and uneven electrode distribution.^33^ Despite these considerations, we used the average reference to maintain consistency with our previous research on male dominance.^34^ Future research will explore alternative referencing techniques, such as the reference electrode standardization technique (REST).

## Resource availability

### Lead contact

Requests for further information and resources should be directed to and will be fulfilled by the lead contact, Wei-Hsiang Lin (weishung1@gmail.com).

### Materials availability

This study did not generate new unique reagents.

### Data and code availability

• All behavioral and EEG data have been deposited in The Open Science Framework (OSF) and are publicly available at https://doi.org/10.17605/OSF.IO/DSAKZ.
• All original code has been deposited in The Open Science Framework (OSF) and are publicly available at https://doi.org/10.17605/OSF.IO/DSAKZ.
• Any additional information required to reanalyze the data reported in this paper is available from the lead contact upon request.

## Acknowledgments

This work was supported by grants from the 10.13039/501100001711Swiss National Science Foundation (CR20I3-146431; 10.13039/501100023650NCCR Synapsy).

## Author contributions

Conceptualization and methodology, W.-H.L., J.R.C., C.S., and M.H.H.; data curation: W.-H.L.; funding acquisition, M.H.H.; investigation, W.-H.L.; formal analysis, W.-H.L.; writing – original draft, W.-H.L. and M.H.H.; writing – review and editing, J.R.C., C.S., and M.H.H.; supervision: C.S. and M.H.H. All authors have read and approved the final version of the manuscript.

## Declaration of interests

The authors declare no competing interests.

## STAR★Methods

### Key resources table

**Table:** 

REAGENT or RESOURCE	SOURCE	IDENTIFIER
**Deposited data**		

Behavioral and EEG data	This paper	https://doi.org/10.17605/OSF.IO/DSAKZ

**Software and algorithms**		

MATLAB	MathWorks	https://www.mathworks.com/products/matlab.html
EEGLAB	Swartz Center for Computational Neuroscience	https://sccn.ucsd.edu/eeglab/
Fieldtrip	N/A	https://www.fieldtriptoolbox.org/
JASP	The JASP Team	https://jasp-stats.org
Analysis codes	This paper	https://doi.org/10.17605/OSF.IO/DSAKZ

### Experimental model and study participant details

Please list here under separate headings all the experimental models/study participants (animals, human participants, plants, microbe strains, cell lines, primary cell cultures) used in the study. For each model, provide information related to their species/strain, genotype, age/developmental stage, sex (and gender, ancestry, race, and ethnicity if reported for human studies), maintenance, and care, including institutional permission and oversight information for the experimental animal/human participant study. The influence (or association) of sex, gender, or both on the results of the study must be reported. In cases where it cannot, authors should discuss this as a limitation to their research’s generalizability.

#### Participants

We recruited twenty-six female participants (mean age = 21.54 ± 1.73) from both the Swiss Federal Institute of Technology in Lausanne (EPFL) and the University of Lausanne (UNIL). No ethnicity or racial background data were collected for this study. After signing the informed consent (Commission cantonale d’éthique de la recherche sur l’être humain; 164/14), participants completed a standardized handedness questionnaire.^35^ We then evaluated their visual acuity in both eyes using the Freiburg Visual Acuity Test,^36^ requiring a minimum acuity of 1.0 for both eyes as a requisite for participation (Table S1). As compensation for their participation, they received 30 CHF per hour for EEG sessions, and 25 CHF per hour for behavioral sessions. To ensure uniform cortisol levels, the EEG experiment sessions were scheduled between 1 PM and 7 PM.

### Method details

#### Personality measurements

Before the experiment, participants completed a series of four questionnaires to assess social dominance motivation, state-trait anxiety, and menstrual cycle status. The questionnaires included the Personality Research Form dominance (PRF-D^15^), two aspects of Speilberger’s State-Trait Anxiety Inventory,^37^ and a menstrual cycle status questionnaire. Each was administrated individually through the online experimental platform, Gorilla.^38^ To negate potential order effects, we randomized both the questionnaire sequence and the item order within each.

We gauged participants’ social dominance motivation using the PRF-D, which they completed at least three days prior to the experiment. This 16-item form contains both positive and negative items, utilizing a true/false response format. Speilberger’s State-Trait Anxiety Inventory (STAI) assesses both trait (STAI-T) and state (STAI-S) anxiety via two separate sections, each containing 20 items. Participants respond on a 4-point Likert scale, ranging from 1 (completely disagree) to 4 (completely agree). Scores can vary from 20 (minimal anxiety) to 80 (extreme anxiety).

Lastly, we presented a two-part questionnaire about menstrual cycle status. The first question discerned if participants were on medications or treatments influencing hormone levels. The second estimated the time since their last menstrual cycle, offering responses ranging from less than a week to three weeks.

#### EEG experiment

The present study was conducted based on the protocol of Experiment 5 of da Cruz et al. (2018a).^8^ We presented 20 emotional faces, half male and half female, with happy and sad expressions. Additionally, we used another 20 male faces with angry and neutral expressions. Faces displaying happiness and sadness were sourced from Ekman and Friesen’s Pictures of Facial Affect Series.^39^ The angry and the neutral expressions were derived from FACES,^40^ Radboud Faces Database^41^ and the Karolinska Directed Emotional Faces.^42^ Faces were presented on a grey background and the luminance was below 1 cd/m2. Participants were positioned 50 cm away from the monitor inside a dimly lit Faraday cage. We utilized The Eye Tribe © eye tracker to monitor gaze consistently, and a chin rest was used to stabilize participants’ heads.

At the start of each trial, participants were asked to fixate a cross at the center of the screen, with an interval varying randomly between 0.5 to 1.5 seconds. A face image was then presented for 0.1 seconds at 26° either to the left or right of the center. Participants had up to 3 seconds to identify the depicted emotion using one of two buttons, each held in a different hand. Failure to respond triggered a short buzzer. The trial was repeated at the end of the block.

The experiment comprised two separate conditions with their order randomized for participants. Condition 1 utilized happy and sad faces (Happy vs. Sad) as stimuli, whereas condition 2 featured angry and neutral expressions (Angry vs. Neutral). The association between response side and emotional valence was counterbalanced among participants. Each condition started with a 10-trial practice round, succeeded by five experimental blocks comprising 80 trials each. Instructions were displayed on the screen before each condition began. Participants were instructed to respond as quickly and accurately as possible, and to maintain their gaze on the central fixation cross.

At the end of the experiment, we recorded a 5-minute resting state EEG with participants keeping their eyes closed.

#### EEG recording and data pre-processing

EEG data was acquired using an Active Two system (BioSemi, The Netherlands) equipped with 128 Ag-AgCl sintered active electrodes, all referenced to the common mode sense (CMS) electrode. The cap’s size and placement were individually adjusted to ensure a proper fit for each participant. The cap was positioned so that the A1 electrode came to lie midway between the inion and the nasion, ensuring a balanced electrode coverage of frontal and occipital regions. Electrooculogram (EOG) recording were captured using electrodes placed 1 cm above and below the right eye and 1 cm lateral to the outer canthus. The EEG recording sampling rate was 2048 Hz. Data was downsampled offline to 512 Hz and underwent a comprehensive pre-processing pipeline based on the protocol by da Cruz et al. (2018b).^34^ This included bandpass filtering from 1 to 50 Hz via a 3rd order Butterworth filter, line-noise removal with CleanLine (www.nitrc.org/projects/cleanline), re-referencing to the bi-weight mean estimate of all channels^43^, elimination of bad channels followed by 3D spline interpolation, removal of bad epochs, and artifact removal using independent component analysis (ICA). EEG epochs were extracted from 100 ms pre-stimulus onset (baseline) to 500 ms post-stimulus onset, and averaged epochs for each participant were baseline corrected. For further artifact reduction, we used the Multiple Artifact Rejection Algorithm (MARA^44^), an ICA-based tool, which allowed us to semi-automatically remove potential noise components.

#### Behavioral experiments

A simple reaction time and a Go/NoGo task were performed by the participants (Figure 4). In the simple reaction time task, a grey square appeared either to the left or right of the central cross. The inter-trial interval varied randomly between 0.15 and 1.5 seconds. Participants were instructed to press the spacebar as soon as the square appeared, irrespective of its position. If no response was given within 0.5 seconds, the square disappeared. A practice session of ten trials was given before the experiment proper, comprising 200 stimuli. To avoid fatigue, short breaks were introduced after every 50 trials. The task typically was completed in about 10 minutes.

In the Go/NoGo task, participants were presented with either a square or a triangle, serving distinctly as the Go or NoGo stimulus. Upon the display of the Go stimulus, participants were asked to hit the spacebar within 0.5 seconds. Conversely, they were instructed to refrain from responding when the NoGo stimulus appeared. Maintaining a 70:30 ratio, the Go stimulus was shown in 70% of the trials. Following the same procedure as the simple reaction time task, after a practice block of ten trials, a block of 200 stimuli was presented. Breaks were introduced every 50 trials, and the entire task took approximately 10 minutes.

To prevent any sequence bias, the order of the two tasks was pseudo-randomized, with half of the participants starting with the simple reaction time task and the other half starting with the Go/NoGo task.

### Quantification and statistical analysis

To understand the influence of various factors on the participants’ behavioral performance, we applied mixed-effect models to incorporate potential variables using JASP with version 0.17.3.0. In the model for reaction times, we included accuracy, trait anxiety, experimental conditions, and dominance groups as fixed effects. We also incorporated an interaction term between experimental conditions and dominance groups, with participant number as a random effect. Statistical significance was set at *p* < 0.05 (two-tailed).

#### Global field power analysis

The global field power (GFP) serves as metric for the reference-independent strength of a neuronal response and is calculated by determining the standard deviation of potentials across all electrodes.^45^ For our study, we computed the GFP individually for each participant and each condition. Subsequent analyses involved repeated-measures ANOVAs at each GFP time point, using group (high and low dominance) and condition (Happy vs. Sad, Angry vs. Neutral) as the factors. Notably, we only regarded effects as reliable if they exhibited continuous significance over a span of 10 ms.^46^

#### Distributed Electrical source imaging

Source analysis was conducted by using the Fieldtrip software.^47^ As we did not have fMRI scans for our experiment, we utilized the head and source models provided directly by Fieldtrip. The layout of the electrodes was aligned to the head model to generate a 3D electrode map. Subsequently, the EEG data was reconstructed using the linearly constrained minimum variance (LCMV) beamformer method, as integrated within the Fieldtrip package. Our primary objective was to pinpoint the disparities in source-space activity between the high and low dominance groups, particularly during the instances where the GFP peaks were observed. Hence, we employed two statistical analyses: a 2 × 2 repeated measures ANOVA to assess the main effects of experimental conditions and dominance groups, as well as their interaction. Secondly, an independent t-test was applied to the reconstructed EEG data at each voxel, contrasting the activity levels between the two dominance groups. To correct for multiple comparisons, we used a spatial criterion requiring clusters to contain at least 15 neighboring solution points with significant effects (*p* < 0.05).^8^

#### Frequency and network analysis

In our resting state EEG data analysis, we employed Fieldtrip^47^ to perform frequency analysis using Welch’s method. This approach was applied across the entire 5-minute EEG dataset. The time series was segmented into 4-second windows, with each segment overlapping its neighboring window by 50%. Within each window, a fast Fourier transform was executed using a DPSS (discrete prolate spheroidal sequences) taper. By averaging over these windows, we obtained a comprehensive spectral estimate. The spectral data were then organized into four distinct frequency bands – delta (1-4 Hz), theta (4-8 Hz), alpha (8-12 Hz), and beta (12-30 Hz). The power was quantified for each band and subsequently averaged across all electrodes.

Further, we delved into the functional connectivity in the EEG data within the frequency domain. Leveraging Fieldtrip, we calculated coherence among electrode pairs for each frequency band. To mitigate potential artifacts from volume conduction, we considered only the imaginary part of coherence. The resulting coherence values gave rise to an assessment of network density, which served as a holistic representation of the overall connectivity in the EEG dataset.

For our statistical approach, we conducted a 4 × 2 ANOVA on both the power and network density values. This was aimed to identify the main effects associated with frequency bands and dominance groups, as well as any interaction effects between these two factors.

#### Salivary cortisol analyses

Participants provided three saliva samples during the study. The first immediately after they signed the consent form, the second at the beginning of condition 2, and the third approximately 20 minutes after the end of the second condition. The time interval between each collection was approximately 1 hour. Each collection gathered approximately 0.8 to 1.4 mL of saliva into 10 mL polypropylene tubes, which were then stored in a freezing environment below -20°C until processing. Subsequently, these samples were centrifuged at a speed of 3000 rpm for a duration of 15 minutes under room temperature conditions. The resulting salivary cortisol and testosterone concentrations were measured using an enzyme immunoassay, following the guidelines provided by the manufacturer, Salimetrics. To account for cortisol’s circadian rhythm, we ensured all experimental sessions took place between 1 PM and 7PM.

Cortisol and testosterone reactivity was evaluated by computing the indices for the area under the curve with respect to ground (AUCg) and the area under the curve with respect to increase (AUCi), following the methodology outlined by Pruessner et al. (2003).^48^ Given that the time intervals between each saliva collection were identical, we used the following formula to calculate AUCg:

$${AUC}_{g}=\sum_{i=1}^{n-1}\frac{\left(m_{\left(i+1\right)}+m_{i}\right)}{2}$$

where n is the total number of measurements (in our case, n = 3), and $m_{i}$ is the individual measurement of cortisol or testosterone. We calculated AUCi using the formula:

$${AUC}_{i}={AUC}_{g}-\left(n-1\right)*m_{1}$$

with the same notation as for AUCg.
