# Supplementary material for: Neural markers of social dominance: A female-focused perspective
Source: iScience. 2025 Jul 15;28(8):113109. doi: 10.1016/j.isci.2025.113109 (PMC12344201; doi:10.1016/j.isci.2025.113109)
Supplement: Document S1. Figures S1 and S2 and Table S1 [file mmc1.pdf]

iScience, Volume *28*

Supplemental information

Neural markers of social dominance: A female-focused perspective

Wei-Hsiang Lin, Janir Ramos da Cruz, Carmen Sandi, and Michael H. Herzog

Supplementary Information

	**Total number**
**Number of participants**	26
**Sex (female)**	26
	**Mean ± SD (or ratio)**
**Age (year)**	21.54 ± 1.73
**Handedness (Right:Left)**	22:4
**Binocular visual acuity**	1.65 ± 0.4

Table S1. Demographic Information. Demographic characteristics of the participants recruited for this study. *Table S1 corresponds to the "Participants" section in the STAR Methods.*

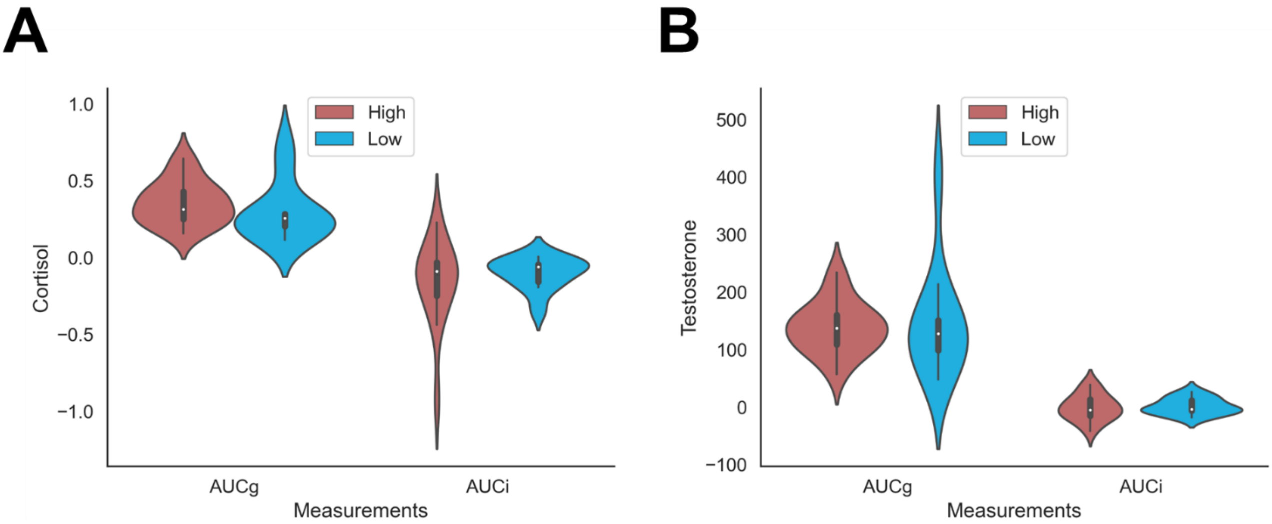

Figure S1. Hormonal levels. (A) Violin plot showing the AUC_g and AUC_i of cortisol of both high and low dominant groups. (B) Violin plot showing the AUC_g and AUC_i of testosterone of both high and low dominant groups. Violin plots in (A) and (B) illustrate the data distribution, with horizontal lines marking the median and quartiles. Further details can be found in the Method: Salivary Cortisol Analyses' section. *Figure S1 corresponds to Figure 1 in the main text.*

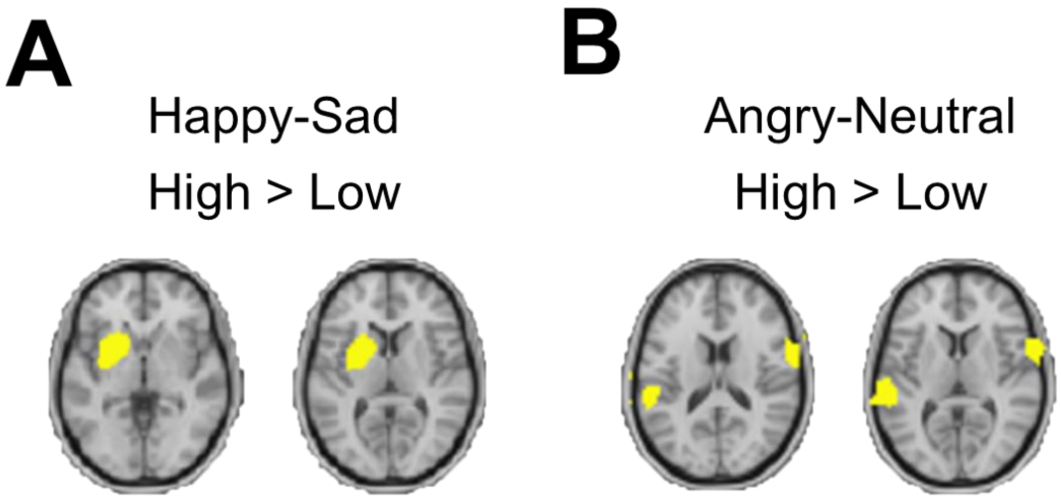

Figure S2. Condition-specific source analysis. (A) The contrast in source space between the high and low dominance groups during the Happy-Sad condition. We applied t-tests at each voxel to compare the high and low dominance groups. The yellow voxels indicate significant differences between the two dominance groups. The two images (left and right) represent the same map at different depths. (B) The contrast in source space between the high and low dominance groups during the Angry-Neutral condition. All other representations are identical to those in (A). *Figure S2 corresponds to Figure 2 in the main text.*
